# Recent Research Progress: Discovery of Anti-Plant Virus Agents Based on Natural Scaffold

**DOI:** 10.3389/fchem.2022.926202

**Published:** 2022-05-26

**Authors:** Jixiang Chen, Xin Luo, Yifang Chen, Yu Wang, Ju Peng, Zhifu Xing

**Affiliations:** ^1^ State Key Laboratory Breeding Base of Green Pesticide and Agricultural Bioengineering, Key Laboratory of Green Pesticide and Agricultural Bioengineering, Ministry of Education, Guizhou University, Guiyang, China; ^2^ Guizhou Rice Research Institute, Guizhou Academy of Agricultural Sciences, Guiyang, China

**Keywords:** plant virus, natural products, antiviral agents, SAR, mechanism

## Abstract

Plant virus diseases, also known as “plant cancers”, cause serious harm to the agriculture of the world and huge economic losses every year. Antiviral agents are one of the most effective ways to control plant virus diseases. Ningnanmycin is currently the most successful anti-plant virus agent, but its field control effect is not ideal due to its instability. In recent years, great progress has been made in the research and development of antiviral agents, the mainstream research direction is to obtain antiviral agents or lead compounds based on structural modification of natural products. However, no antiviral agent has been able to completely inhibit plant viruses. Therefore, the development of highly effective antiviral agents still faces enormous challenges. Therefore, we reviewed the recent research progress of anti-plant virus agents based on natural products in the past decade, and discussed their structure-activity relationship (SAR) and mechanism of action. It is hoped that this review can provide new inspiration for the discovery and mechanism of action of novel antiviral agents.

## 1 Introduction

Plant viruses are a serious threat to the safe production of world agriculture, causing global economic losses as high as $60 billion every year (Bos, 2000; Barna et al., 2003; Zhao et al., 2017a). Tobacco mosaic virus (TMV), tomato spotted wilt virus (TSWV), tomato yellow leaf curl virus (TYLCV), cucumber mosaic virus (CMV), potato virus Y (PVY) are the top five most important plant viruses of the world according to science/economic importance (Scholthof et al., 2011). TMV is one of the oldest known plant viruses and ranks first among the top 10 plant viruses, causing economic losses in excess of $100 million per year. The host range of TMV exceeds 400 species, and TMV may alter the metabolism and impair the defense system of hosts (Silverman et al., 2005; Scholthof et al., 2011; Sharma et al., 2021). After the plant virus invades the host, the substances required for the life process are completely dependent on the host, and its replication may be combined with the metabolism of the host, making it difficult to prevent and control the viral diseases (Rodrigues et al., 2016; Wang D. et al., 2021). There is no antiviral agent that can completely inhibit plant viruses, and the development of high-efficiency antiviral agents still faces huge challenges (Gan et al., 2021).

**FIGURE 1:**
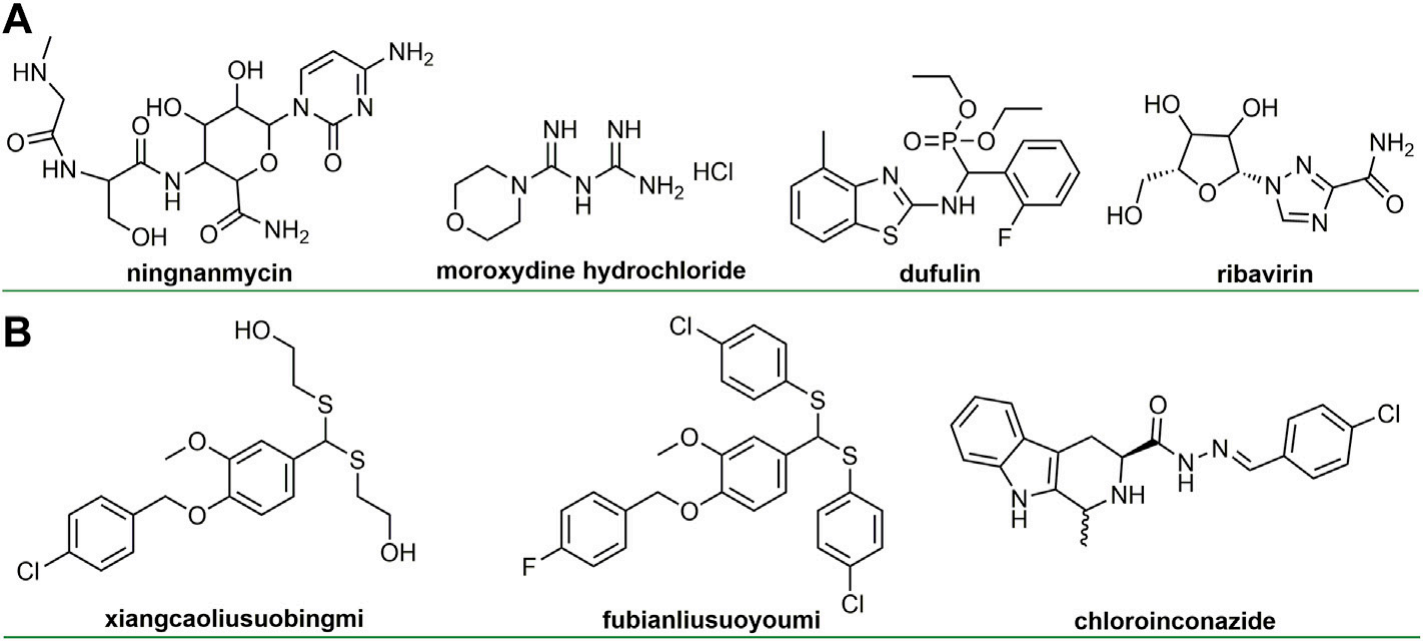
Chemical structures of commercialized **(A)** or under development antiviral agents **(B)**.

Natural products have long been regarded as a source of inspiration for drug design, providing many unknown chemical scaffolds and pharmacophores (Eschenbrenner-Lux et al., 2014). In addition, natural products readily interact with biological targets, thereby exhibiting specific biological activities (Lowe, 2014; Bauer and Brӧnstrup, 2014). Identifying natural product structure and studying biological activity are of great significance for drug discovery (Chen and Song, 2021; Della-Felice et al., 2022). The discovery of anti-plant virus agents based on natural products is an important research direction in the prevention and control of plant virus diseases and has always attracted much attention (Eckert et al., 2003; Carli et al., 2012; Katayama et al., 2013; Wang et al., 2015). Ningnamycin (Figure 1A), isolated from *Strepcomces noursei* var *xichangensisn* for the first time, has broad-spectrum and excellent antiviral activity and is currently the most successful antiviral agent, playing a huge role in the control of plant virus diseases (Han et al., 2014). Ningnanmycin promotes the accumulation of pathogen-related proteins (PRs), a marker of systemic acquired resistance (SAR), by inhibiting the polymerization process of TMV coat protein (TMV-CP) (Han et al., 2014). In addition, ningnamycin can activate redox and metabolic processes in CMV-infected tobacco (Gao et al., 2019).

In recent years, great progress has been made in the research and development of anti-plant virus agents (Carli et al., 2010; Li and Song, 2017; Park et al., 2021). The discovery of some new antiviral agents based on natural products (Figure 1B) not only reflects the important role of natural products in the discovery of antiviral agents, but also provides great help for the control of plant viruses. Special natural molecular scaffolds can serve as bridges for the derivatization of antiviral agents (Jassbi et al., 2017; Chen J. et al., 2020), which can provide innovative solutions for the discovery of novel antiviral agents. We wish to analyze the research progress of chemical antiviral agents based on natural scaffold. However, most of these references come from China in recent 10 years. From the perspective of natural products, we reviewed the latest research progress of anti-plant virus chemical active compounds in recent years and discussed their anti-viral activity, structure-activity relationship and mechanism of action, aiming to provide new insights for the discovery of new anti-viral agents.

## 2 Antiviral Active Compounds

### 2.1 Acids

#### 2.2.1 Fatty Acids or Carboxylic Acids

**FIGURE 2:**
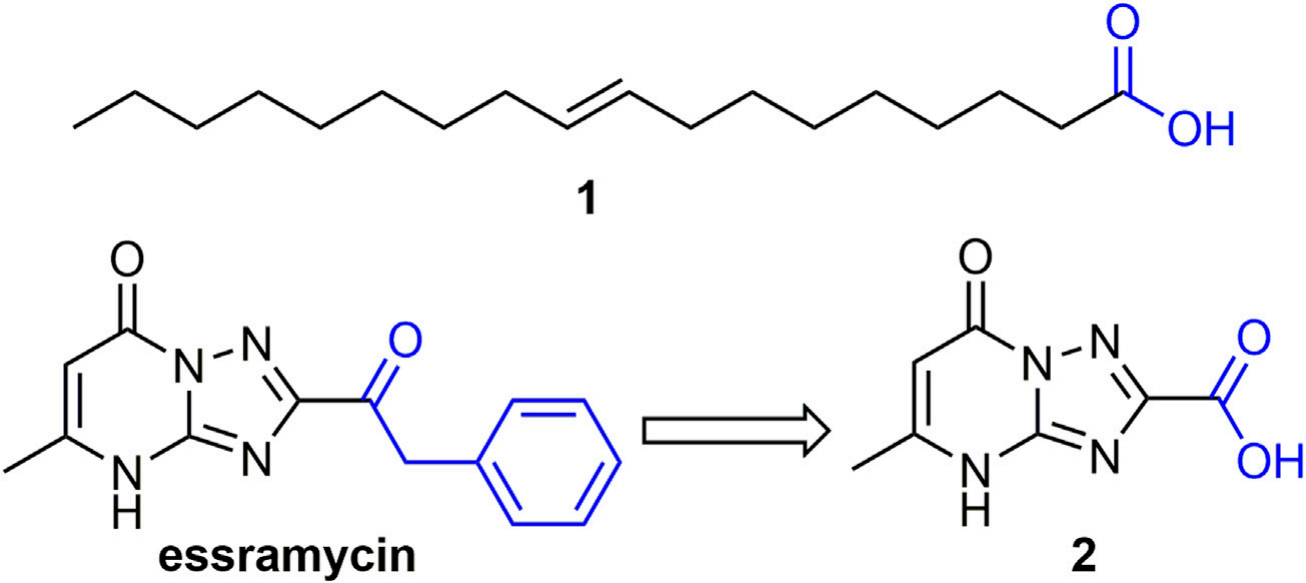
Structures of representative fatty acids or carboxylic acids in antiviral activities.

Some natural fatty acids or carboxylic acids have good anti-plant virus activity (Katayama et al., 2013; Deshoux et al., 2020). For example, compound **1** (Figure 2), isolated from cottonseed sludge was able to increase the phenylalanine ammonia lyase (PAL) and peroxidase (POD) activities of tobacco, as well as the expression levels of *PR-1a* and *PR-5* genes. Its anti-plant virus activity may be related to the expression and activation of various defense-related genes in tobacco (Zhao et al., 2017b). Compound **2**, a derivative of the marine natural product essramycin, exhibited 62, 64, and 68% of TMV inactivating, curative, and protective activities at 500 mg/L, respectively. Compound **2** showed antiviral activity by inhibiting viral assembly and promoting aggregation of 20S disk proteins (Wang T. et al., 2020).

#### 2.2.2 Ferulic Acid Derivatives

**FIGURE 3:**
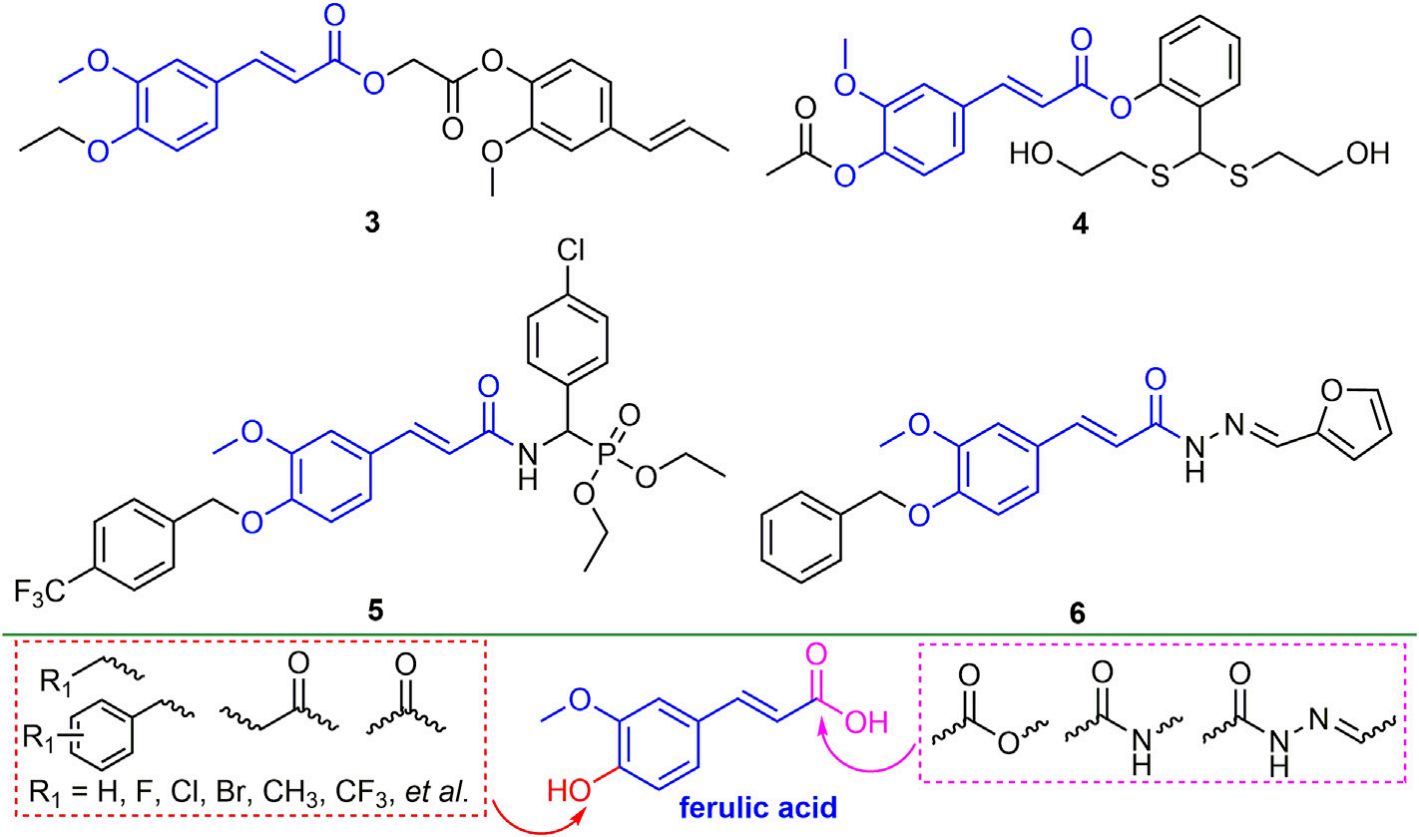
Structures of representative ferulic acid derivatives in antiviral activities.

Ferulic acid is widely found in plants, and its derivatives have broad-spectrum biological activities (Sonar et al., 2019; Boulebd et al., 2022). Ferulic acid derivatives have good performance in antiviral activity. For example, compound **3** (Figure 3) has EC_50_ values of 135.5 and 178.6 mg/L for TMV and CMV. Compound **3** can significantly alter the levels of tobacco gene transcription and protein expression, and enhance the defense response of tobacco by inducing the accumulation of secondary metabolites in the biosynthetic pathway of tobacco phenylpropanoid, thereby inhibiting virus infection (Gan et al., 2021). At a concentration of 500 mg/L, the curative, protective and inactivating activities of compound **4** against TMV were 62.5, 61.8 and 83.5%, respectively. Compound **4** is not only able to cause the breaking and bending of TMV, but also has a strong binding force on TMV-CP (Wang Y. et al., 2020). The EC_50_ values for the curative and protective activity of compound **5** against CMV were 284.67 and 216.30 mg/L, respectively (Lan et al., 2017). The EC_50_ value of compound **6** for TMV inactivating activity was 36.59 mg/L (Wang et al., 2017). The derivatization of ferulic acid is mainly phenolic hydroxyl and carboxyl moieties. In the phenolic hydroxyl part, benzyl, alkyl, and carbonyl groups are mainly introduced for derivatization, and in the carboxyl part, new ferulic acid derivatives are synthesized mainly through esterification, amidation and acylhydrazone.

### 2.2 Ketones

#### 2.2.1 Chalcone Derivatives

**FIGURE 4:**
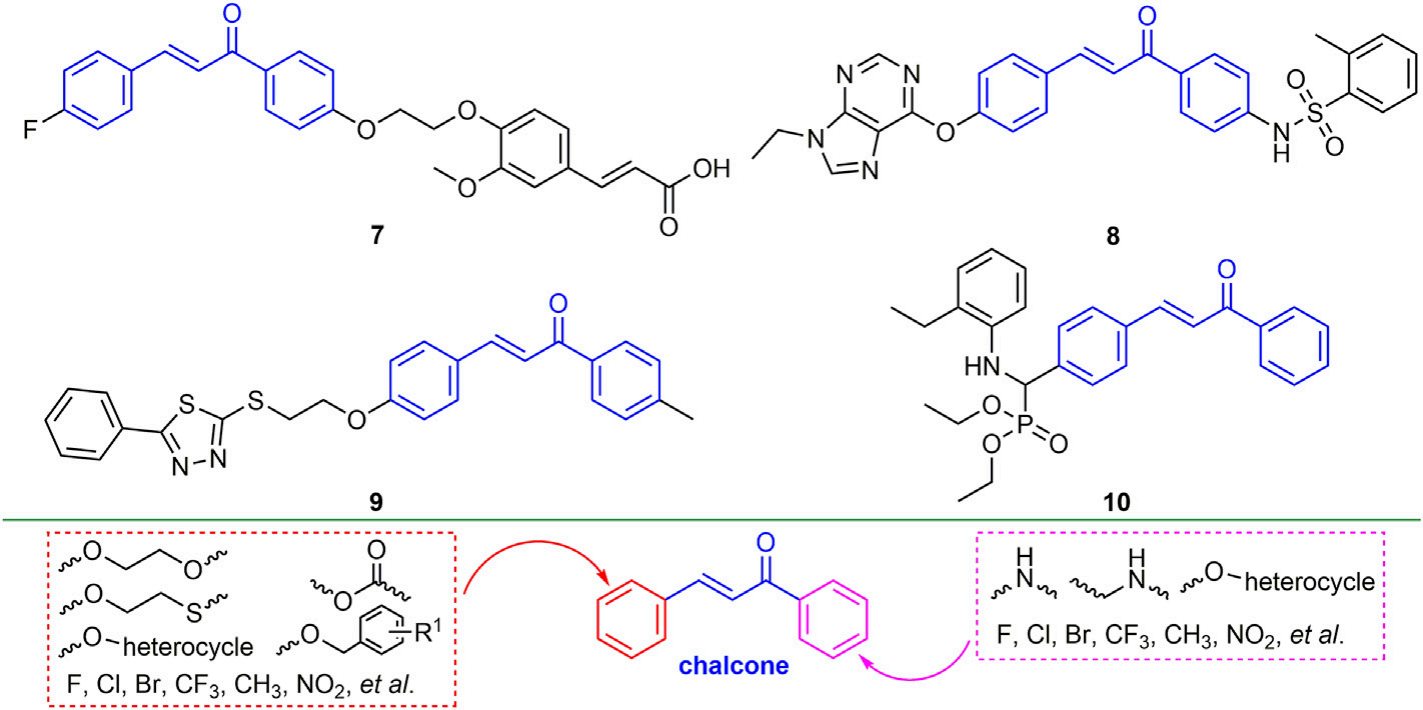
Structures of representative chalcone derivatives in antiviral activities.

Chalcone derivatives showed good antiviral activity (Sinha, et al., 2019). For example, compound **7** (Figure 4) showed 55.6, 71.2 and 92.4% of curative, protective, and inactivating activities against TMV, respectively. Compound **7** induced plant tolerance to mosaic virus by enhancing tobacco defense enzyme activity, chlorophyll content, and photosynthesis (Gan et al., 2017a). Compounds **8** and **9** not only have good passivation activities (EC_50_, 51.65 and 30.57 mg/L) for TMV, but also have a strong binding ability to TMV-CP (Gan et al., 2017b; Zhou et al., 2018). Compound **10** has good curative and protective activities against TMV (57.6 and 59.9%), which may trigger the breakdown of TMV by directly interacting with TMV, while also inducing plant resistance (Zhou et al., 2021). The derivatization direction of chalcone is mainly two benzene rings. The benzene ring connected to the carbonyl group mainly introduces halogen, alkoxy, alkyl, and aryl ether, while the benzene ring connected to the double bond mainly introduces halogen, alkoxy, benzyl, aryl ether and ester groups.

#### 2.2.2 Pentadienone Derivatives

**FIGURE 5:**
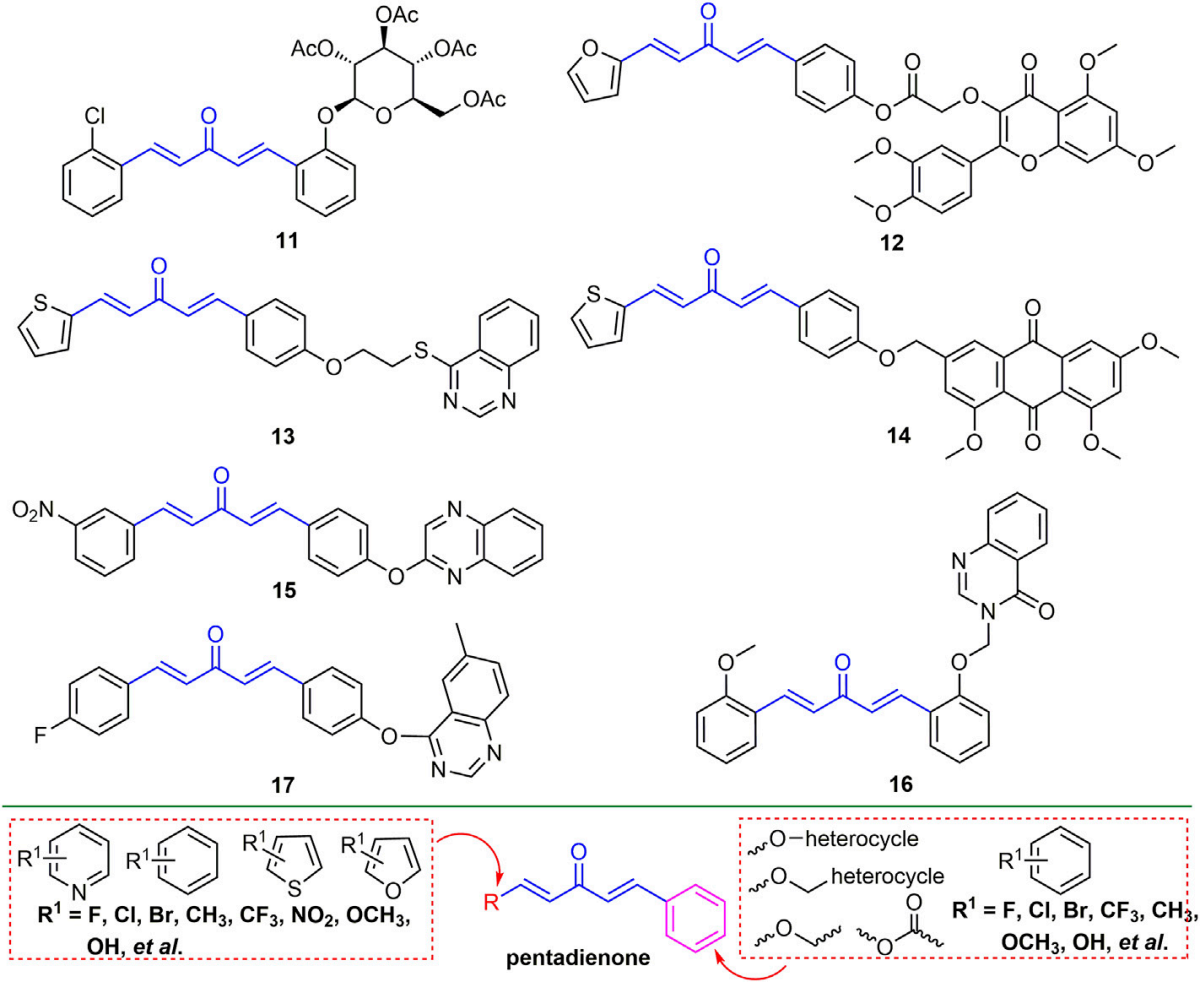
Structures of representative pentadienone derivatives in antiviral activities.

There have been many reports on the antiviral activity of pentadienone derivatives. For example, compound **11** (Figure 5) has an EC_50_ value of 52.9 mg/L for TMV inactivation activity and has a micromolar affinity for TMV-CP (Chen et al., 2015). At 500 mg/L, the *in vivo* curative activity of compound **12** against TMV and CMV was 48.2 and 59.84%, respectively, and the inactivation activity was 82.6 and 89.8%, respectively (Han et al., 2015). The EC_50_ values of the protective activity against TMV and the curative activity against CMV of compound **13** were 124.3 and 365.5 mg/L, respectively (Long et al., 2015). At a concentration of 500 mg/L, the curative and protective activities of compound **14** against TMV were 52.6 and 55.4%, respectively (Wu et al., 2016). Compound **15** has a strong binding affinity to CMV-CP with a dissociation constant of 0.071 *μ*Μ (Li et al., 2019). The EC_50_ value of compound **16** for TMV curative activity was 132.2 mg/L (Ma et al., 2014). The EC_50_ values of compound **17** for the curative, protective and inactivating of TMV were 441.3, 364.6, 243.3 mg/L, and the EC_50_ values for the curative, protective and inactivating activity of CMV were 533.6, 490.7 and 471.6 mg/L, respectively (Luo et al., 2013). The backbone structure of 1,4-pentadien-3-one was obtained by curcumin derivatization. The modification of the 1,4-pentadien-3-one structure is mainly at the terminal positions of the two olefinic bonds. If one of the positions is a benzene ring, halogen, alkoxy, benzyl, and ester groups are mainly introduced into the benzene ring. And another position can be benzene ring, thiophene, furan, and pyridine ring, and mainly halogen is introduced in the ring.

#### 2.2.3 Quinazolinone Derivatives

**FIGURE 6:**
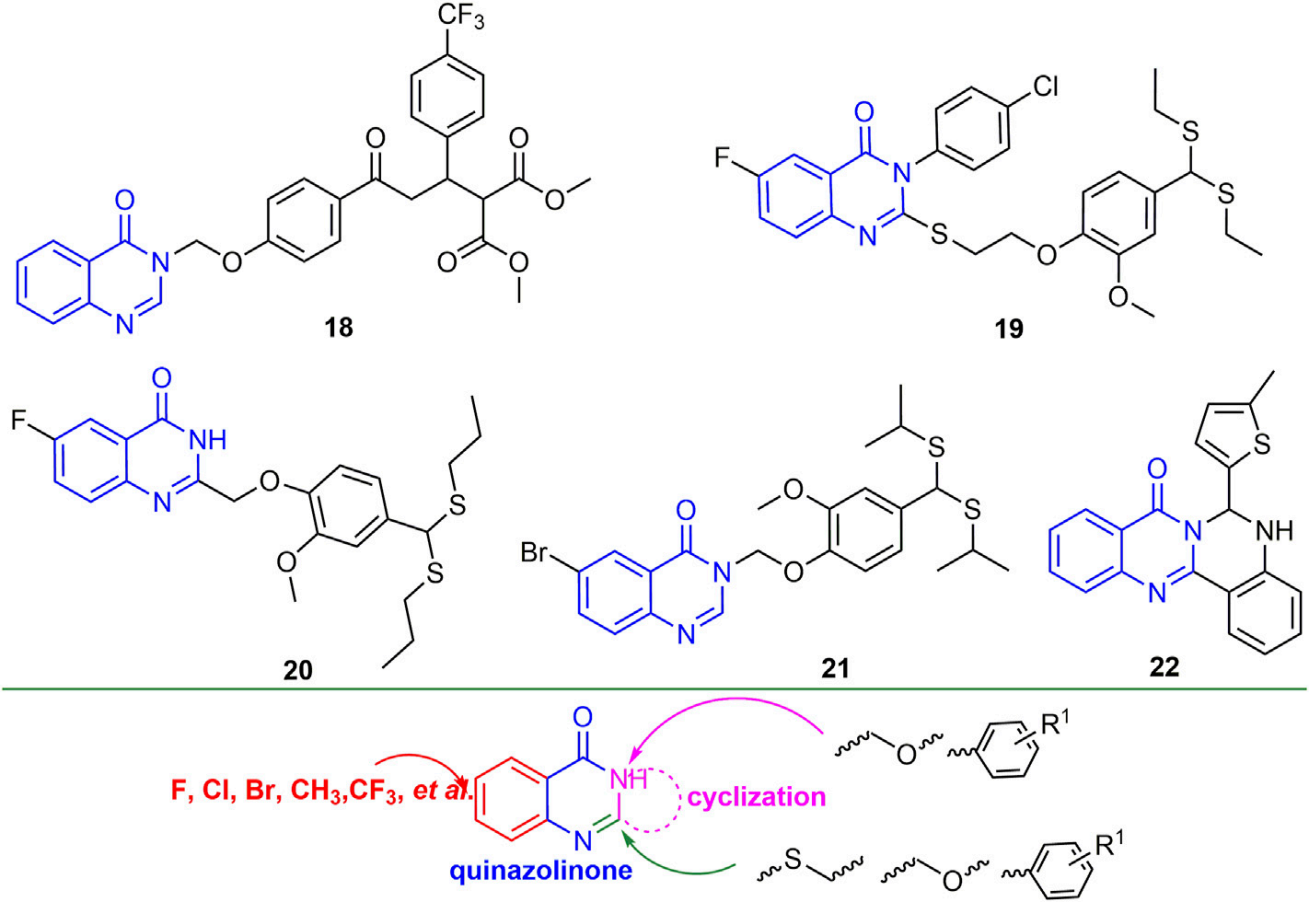
Structures of representative quinazolinone derivatives in antiviral activities.

Quinazolinone is the backbone structure of many alkaloids, and its derivatives have good anti-plant virus activity. For example, compound **18** (Figure 6) not only exhibited good curative activity against CMV (EC_50_, 146.30 mg/L), but also had micromolar binding to CMV-CP (Chen L. et al., 2016). Compounds **19** and **20** have a strong binding ability to tomato chlorosis virus coat protein (ToCV-CP), and the relative expression of ToCV-CP gene in tomato was decreased by 93.3 and 81.0%, respectively (Ran et al., 2020; Zu et al., 2020). Compound **21** not only has good inactivating activity against TSWV (EC_50_, 188 mg/L), but also has a strong binding force to TSWV coat protein (Liu et al., 2021). At 500 mg/L, the inactivating, curative and protective activities of compound **22** against TMV were 51, 43 and 54%, respectively. In addition, compound **22** may show excellent antiviral activity by preventing viral assembly (Hao et al., 2020). Halogen, CF_3_, and alkyl are mainly introduced into the benzene ring of quinazolinone. The two- and 3-positions of quinazoline are mainly introduced into thioether, ether and benzene rings, while between the two- and 3-positions, new heterocycles can be obtained by cyclization.

#### 2.2.4 Chromone Derivatives

**FIGURE 7:**
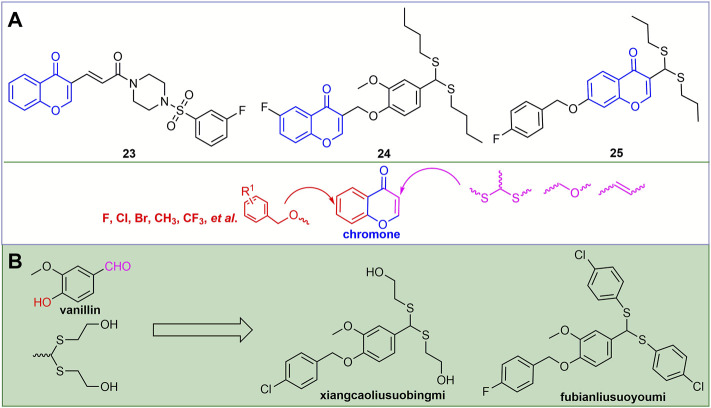
Structures of representative chromone derivatives in antiviral activities **(A)**. Discovery of novel antiviral agents xiangcaoliusuobingmi and fubianliusuoyoumi **(B)**.

Chromones, which are widely present in plants, have good antiviral activity. For example, compound **23** (Figure 7A) not only has good binding ability to ToCV-CP, but also reduces the relative expression of ToCV-CP gene by 67.2% (Jiang et al., 2021). The curative and protective EC_50_ values of compound **24** against TSWV were 124.2 and 109.3 gm/L, respectively. It may exert antiviral activity by blocking the binding of TSWV N to viral RNA (Zan et al., 2021). At a concentration of 500 mg/L, compound **25** exhibited good curative, protective and inactivating activities against TMV, which were 68.8, 58.8, and 86.0%, respectively. It may exert antiviral activity by disrupting the phenotype and integrity of TMV (Li et al., 2020). Halogen, CF_3_, alkyl and benzyl are mainly introduced into the benzene ring of chromone. The second-flow acetal was mainly introduced at the 3-position of the chromone, and the oxygen-containing flexible chain and the olefinic bond were derivatized.

### 2.3 Vanillin Derivatives

Vanillin is a natural fragrance with a strong aroma and is widely used in the production of cosmetics and fragrances (Libardi et al., 2011; Kayaci and Uyar, 2011). For example, the discovery of novel antiviral agents xiangcaoliusuobingmi and fubianliusuoyoumi (Figure 7B) benefits from this. The EC_50_ values of xiangcaoliusuobingmi for the curative and protective activities against PVY were 217.6 and 205.7 mg/L, respectively, and the EC_50_ values for the curative and protective activities against CMV were 206.3 and 186.2 mg/L, respectively (Zhang et al., 2017). In addition, it can regulate the expression of defense genes and increase the activity of defense enzymes to exert antiviral activity (Shi et al., 2018).

**FIGURE 8:**
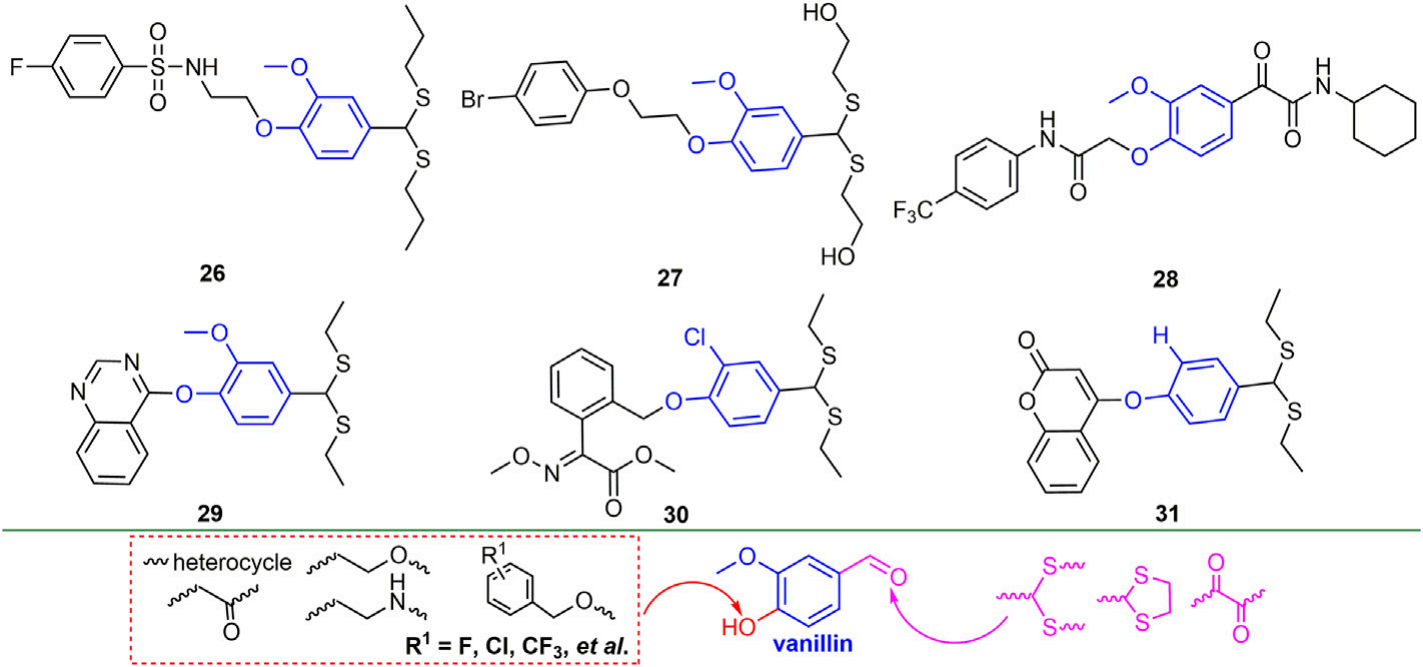
Structures of representative vanillin derivatives in antiviral activities.

The EC_50_ for the curative, protective and inactivating activities of compound **26** (Figure 8) against TMV were 329.5, 269.2 and 48.1 mg/L (Yang Y. et al., 2020). The curative, protective and inactivating activities of compound **27** against TMV were 50.9, 58.9 and 81.8%, respectively. Compound **27** can not only destroy the morphology of TMV particles, but also has a strong binding effect with TMV-CP (Wang et al., 2019). At 500 mg/L, compound **28** exhibited 51.8 and 90.1% of the curative and inactivating activities against TMV, respectively, and it was able to hinder the self-assembly of TMV (Luo et al., 2020). Compound **29** inhibited ToCV infection in the host and decreased the expression level of ToCV-mCP gene (Yang H. et al., 2020). Compound **30** has good curative and protective activities against PVY, CMV and TMV, and can improve the resistance of tobacco to viruses (Chen et al., 2018).

The curative, protective and inactivating activities of compound **31** against TMV were 62.1, 54.5 and 94.2%, respectively, and it could disrupt the structure of TMV particles, thereby inhibiting virus infection (Zhao et al., 2020a; Zan et al., 2020). The modification sites of vanillin are mainly hydroxyl and aldehyde groups, and benzyl, alkoxy, imino, heterocyclic and carbonyl groups are mainly introduced into the hydroxyl part, while the aldehyde groups are mainly changed to dithioacetal and carbonyl. Among them, the antiviral activity was significantly improved after the aldehyde group was changed to dithioacetal, and the chain length and substituent of the acetal also significantly affected the antiviral activity of the compounds.

### 2.4 Indole Derivatives

**FIGURE 9:**
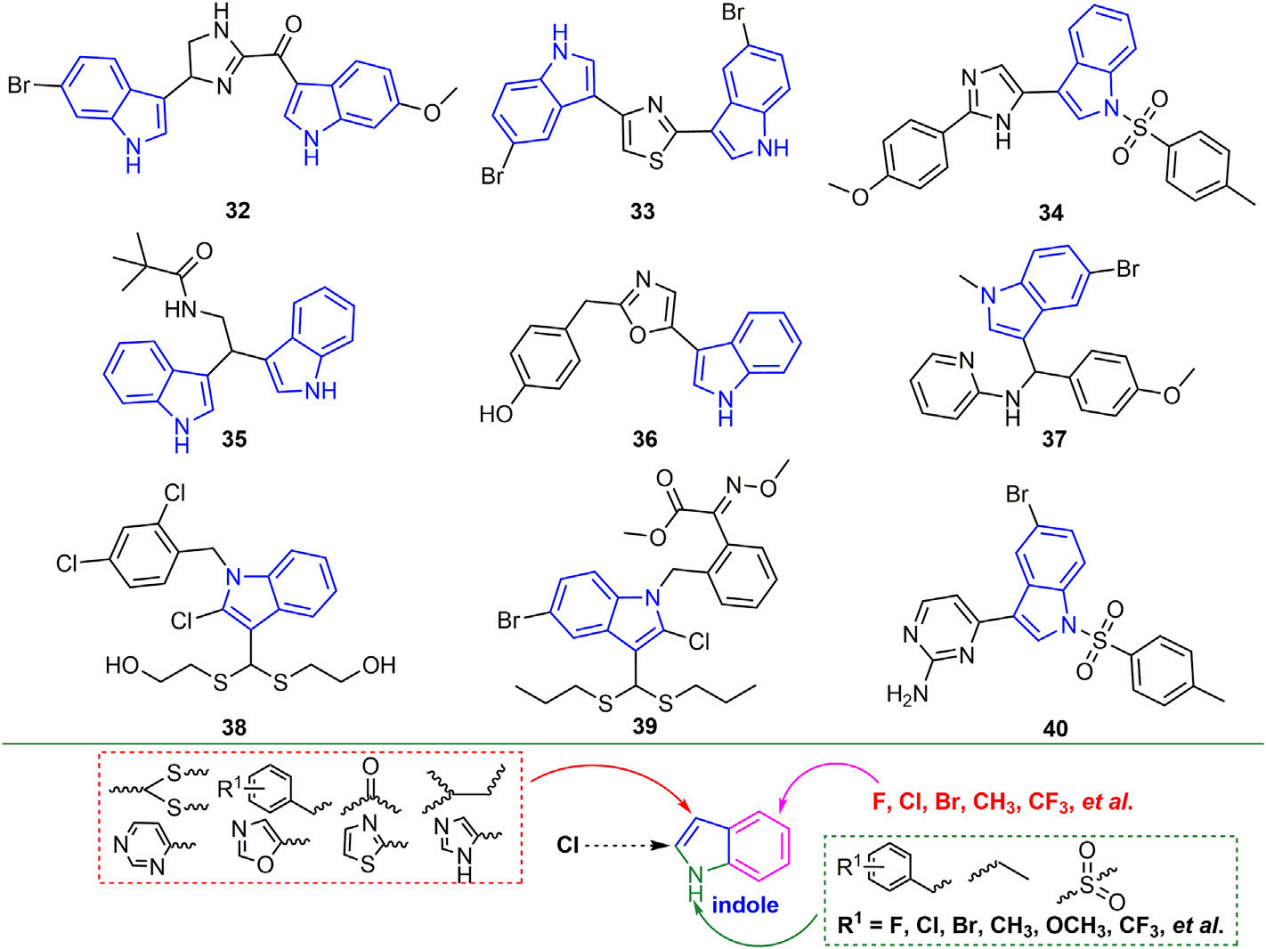
Structures of representative indole derivatives in antiviral activities (I).

Indole is the core backbone structure of many alkaloids and is widely used in the discovery of pesticides (Çokuğraş and Bodur, 2013; Ji et al., 2016; Rajasekharan et al., 2020; Dong et al., 2020). The inactivating, curative and protective activities of compound **33** against TMV were 54, 50, and 53%, respectively, and it may exert antiviral activity by preventing the movement of the virus in plants (Figure 9) (Guo et al., 2019). Compound **35** may show good anti-TMV activity by inhibiting the assembly of viral particles and the aggregation of 20S CP (Kang et al., 2020). The antiviral activity of compound **39** against TMV, CMV, and PVY was associated with an increase in chlorophyll content and defense-related enzyme activity (Wei et al., 2019). Antiviral activity of other representative indole derivatives is shown in Table 1. Halogen, alkyl and CF_3_ are mainly introduced into the benzene ring of indole. Benzyl, alkyl and sulfonyl groups are mainly introduced on the N atom. At the 3-position, heterocycle, benzene ring, dithioacetal, carbonyl and alkyl are mainly introduced for derivatization.

**TABLE 1:** Antiviral activity of other representative indole derivatives.

Comp	Anti-TMV Activity				Mechanism	Ref
	Concentration (mg/L)	Curative (%)	Protective (%)	Inactivating (%)		
35	500	50	53	54	—	Ji et al. (2018)
37	500	56	52	55	—	Liu et al. (2019)
38	500	65	66	68	Possibly inhibiting viral assembly by cross-linking TMV-CP.	Lu et al. (2019)
39	500	55.1	57.2	80.3	Not only destroys the TMV particle morphology, but also has a strong interaction with TMV-CP.	Wei et al. (2020)

**FIGURE 10:**
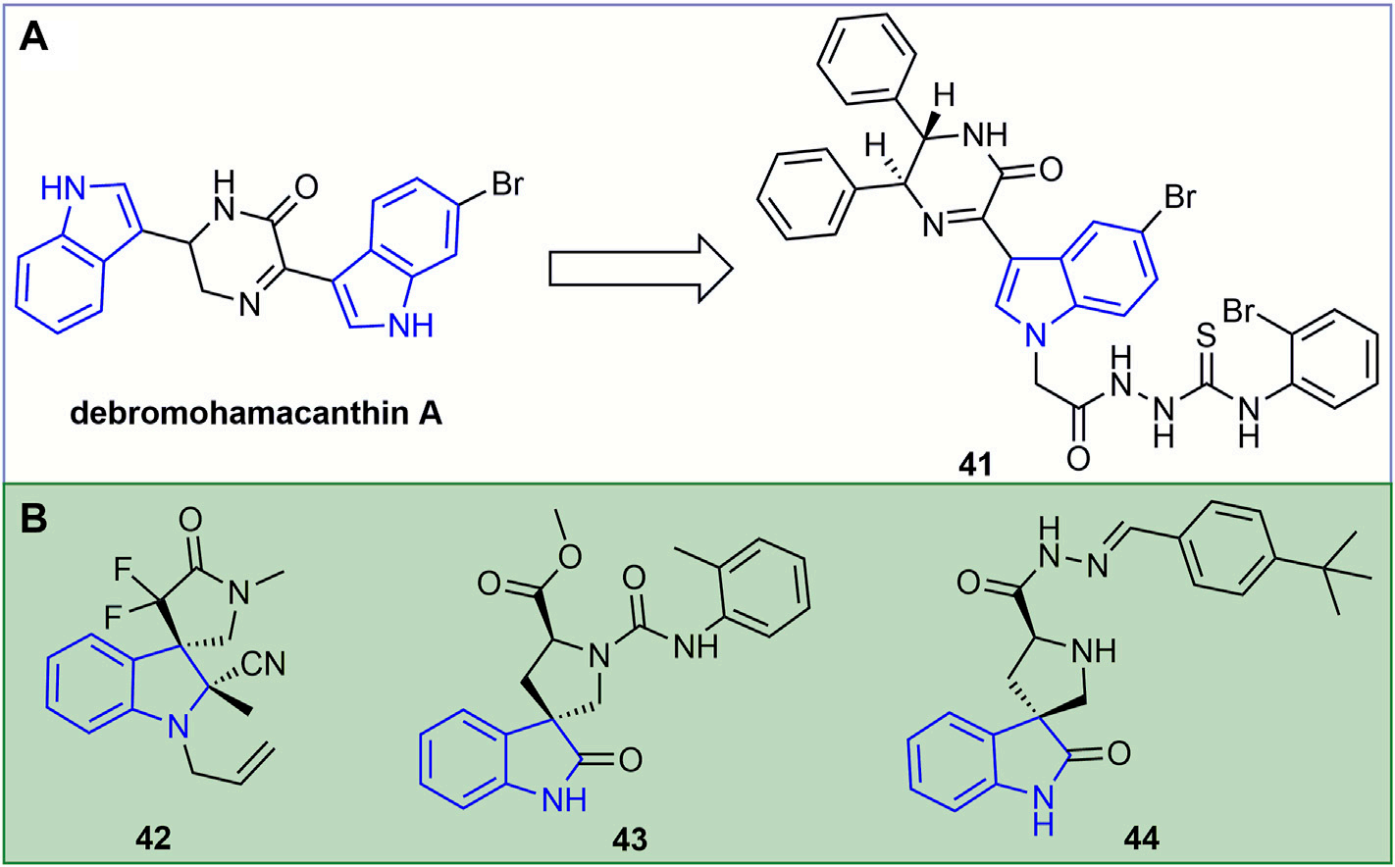
Structures of representative of debromohamacanthin A derivatives in antiviral activities **(A)**. Structures of representative of indole spirocycles derivatives in antiviral activities **(B)**.

The inactivating, curative, and protective activities of the marine natural product debromohamacanthin A (Figure 10A) against TMV were 53, 51 and 56%, respectively. The inactivating, curative, and protective activities of its derivative **41** against TMV were 60, 59, and 63%, respectively, and the antiviral activity of the compound was improved through structural optimization (Wang T. et al., 2021). In addition, compound **41** can bind to TMV-CP and interfere with the assembly process of TMV-CP and RNA, thus showing antiviral resistance. The inactivating, curative, and protective activities of compound **42** (Figure 10B) against TMV were 51.2, 49.0, and 53.6%, respectively (Wang et al., 2022). The functional groups containing CF_2_, indole or cyano favored the antiviral activity of 3,3-helix cyclic indole derivatives. At 500 mg/L, the curative, protective, and inactivating activities of compound **43** against TMV were 47, 50, and 51%, respectively (Chen L. et al., 2020). At 500 mg/L, the inactivating, curative, and protective activities of compound **44** were 58, 55.2, and 49.7%, respectively (Chen M. et al., 2016).

**FIGURE 11:**
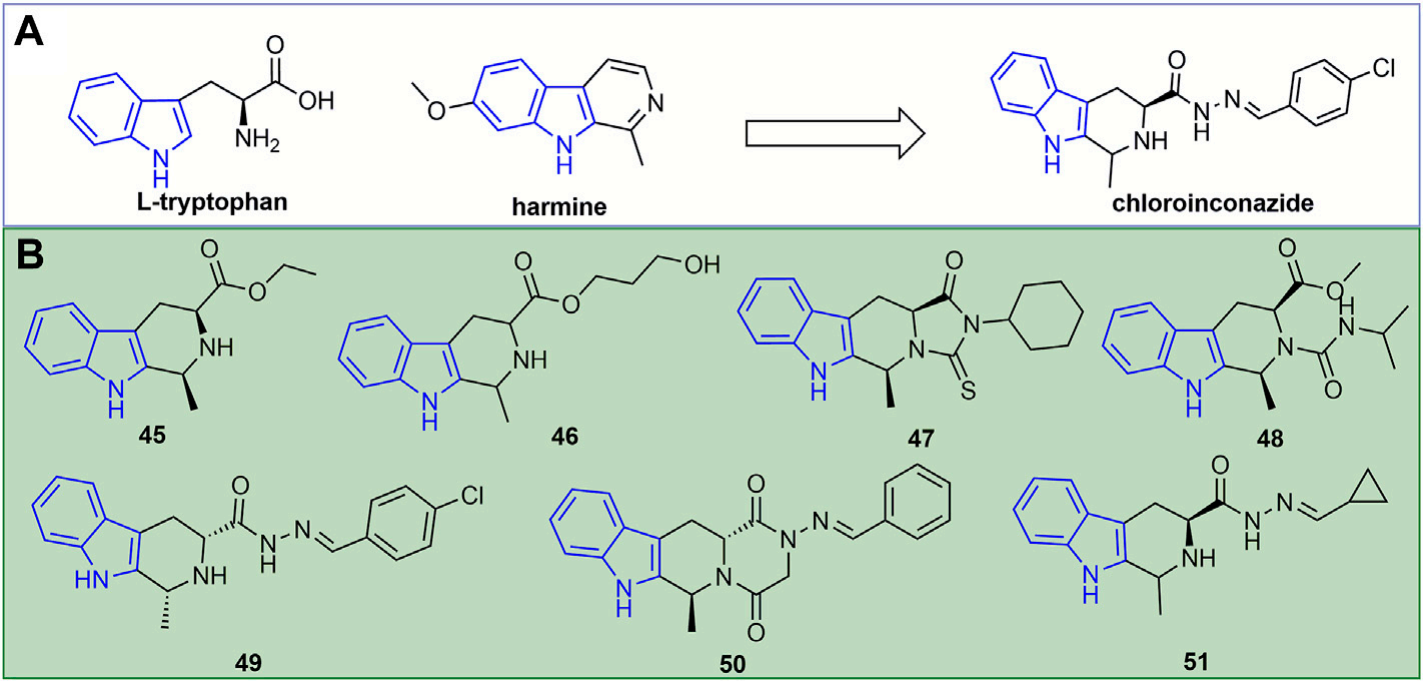
Discovery of a novel antiviral agent chloroinconazide **(A)**. Structures of representative indole derivatives in antiviral activities **(B)**.

A novel antiviral agent, chloroinconazide (Figure 11A), was discovered based on the natural product harmine. Its inactivating, curative, and protective activities at 500 mg/L against TMV were 70.4, 71.5, and 64.2%, respectively (Liu et al., 2014). Chloroinconazide can also activate reactive oxygen species and antioxidant levels, induce an increase in salicylic acid content and the expression of its response gene *PR2* (Lv et al., 2021). Furthermore, it can attenuate the virulence of TMV by directly changing the morphological structure of the virion and increasing the activity of antioxidant enzymes, thereby reducing the production of TMV-induced reactive oxygen species (ROS) during plant infection (Lv et al., 2020).

At 500 mg/L, the inactivating, curative, and protective activities of compound **46** (Figure 11B) against TMV were 50.4, 43.9, and 47.9% (Song H. et al., 2014). The *in vitro* antiviral activity of compound **47** against TMV was 48.2% (Song H.-j. et al., 2014). The anti-TMV inactivating, curative, and protective activities of compound **48** were 59, 63 and 60% at 500 mg/L, respectively (Huang et al., 2018). Compound **49** showed excellent inactivating, curative, and protective activities against TMV with EC_50_ values of 127, 156, and 108 mg/L, respectively (Wang and Song, 2020c). Compound **50** not only retarded TMV proliferation, but also had a concentration-dependent effect on tobacco growth and biomass accumulation (Zhang X. et al., 2021). The inactivating, curative, and protective activities of compound **51** against TMV were 49, 50 and 52%, respectively (Xie et al., 2020).

### 2.5 Tylophorine Derivatives

**FIGURE 12:**
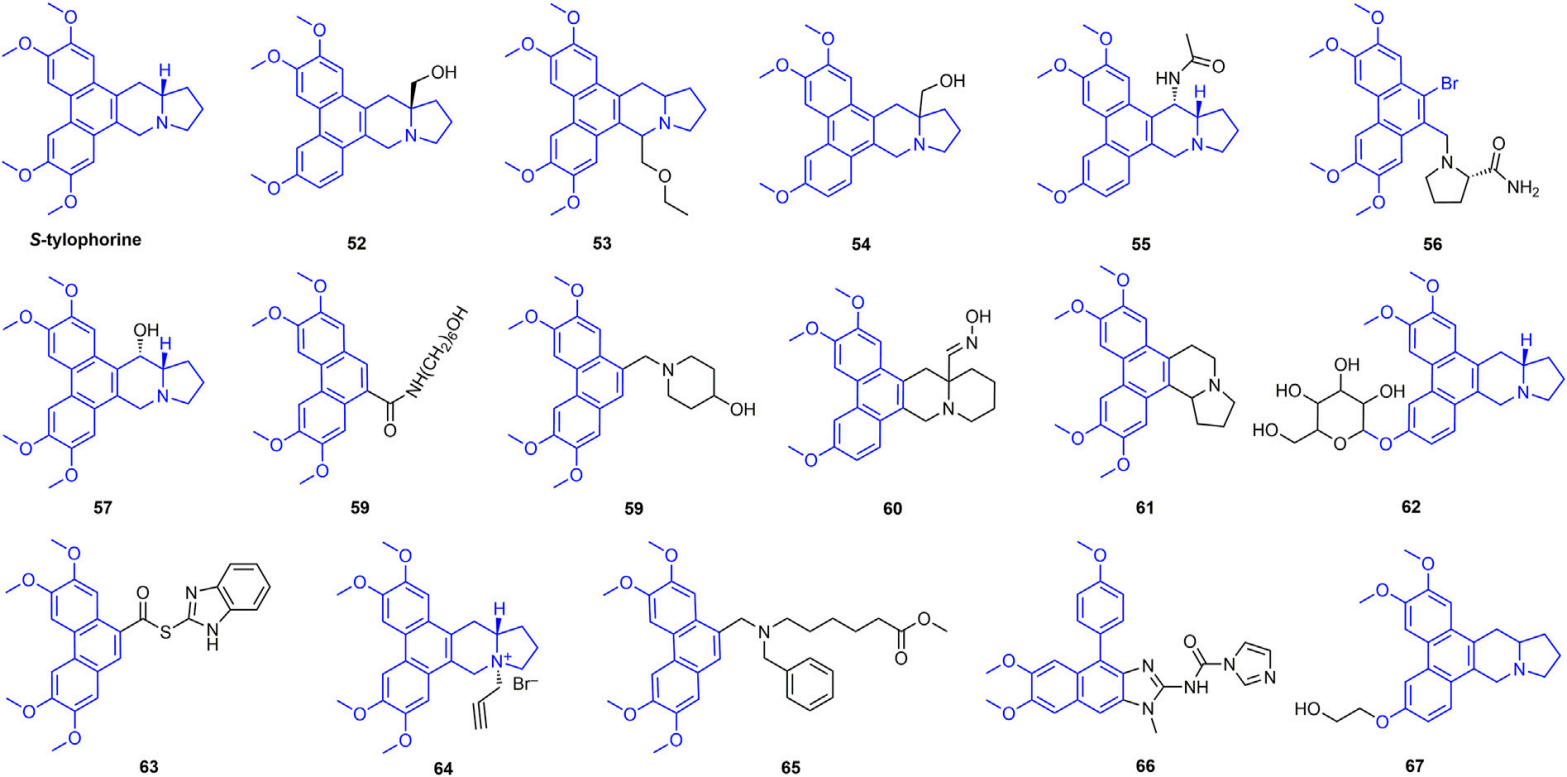
Structures of representative tylophorine analogues in antiviral activities.

Tylophorine has good antiviral activity, and there have been many reports on the antiviral activity of its derivatives (Figure 12) (Wang et al., 2010a). For example, the inactivating, curative, and protective activities of compound **52** against TMV were 78.1, 80.1 and 88.4%, respectively. The planarity of the molecule and the rigidity of the D-ring also have a strong effect on the activity, suggesting that the three-dimensional conformation is also very important for enhanced biological activity (Su et al., 2016). At 500 mg/L, the inactivating, curative, and protective activities of compound **53** against TMV were 67.7, 65.3, and 65.9%, respectively, and its EC_50_ value was 296 mg/L (Yan et al., 2021). The inactivating, curative and protective activities of compound **54** against TMV were 75.3, 76.2 and 68.4% at 500 mg/L, respectively. The methoxy group on the phenanthrene unit significantly affects the antiviral activity of the compounds (Su et al., 2014a). Antiviral activity of other representative tylophorine analogues is shown in Table 2.

**TABLE 2:** Antiviral activity of other representative tylophorine analogues.

Comp	Anti-TMV Activity (500 mg/L)				Ref
	Concentration (mg/L)	Curative (%)	Protective (%)	Inactivating (%)	
55	500	69.6	72.7	72.2	Wang et al. (2012a)
56	500	36.6	39.5	42.1	Wang et al. (2012b)
57	500	67.9	63.7	57.8	Wang et al. (2012c)
58	500	81	84	-	Wang et al. (2010b)
59	500	37.4	70.8	71.1	Wang et al. (2014a)
60	500	65.8	69.2	70.3	Su et al. (2021)
61	500	58.6	54.1	55.8	Su et al. (2014b)
62	500	68.1	69.3	63.2	Wu et al. (2014)
63	500	56	53	57	Yu et al. (2016)
64	500	82.1	77.6	76.6	Han et al. (2018)
65	500	49.4	55.3	52.6	Wang et al. (2014b)
66	500	66	71	68	Li et al. (2018)
67	500	44	42.3	40.5	Wu et al. (2013)

### 2.6 Purine Nucleoside Derivatives

**FIGURE 13:**
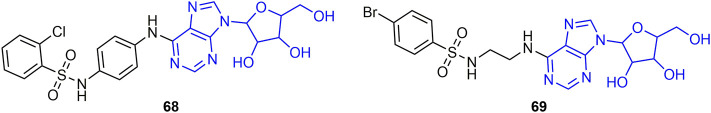
Structures of representative purine nucleoside derivatives in antiviral activities.

Purine nucleosides have excellent antiviral activity, and the curative and protective activities of its derivative **68** (Figure 13) against PVY and CMV were 52.5, 60.0, 60.2%, respectively. The excellent antiviral activity of compound **68** is related to its immune-inducing effect, which can regulate the activities of defense-related enzymes, defense-related genes, and photosynthesis-related proteins in plants (He et al., 2019). The EC_50_ values of the protective activity of compound **69** against CMV and PVY were 137 and 209 mg/L, respectively. The EC_50_ value of compound **69** for the inactivating activity of TMV was 48 mg/L. Compound **69** may further damage the viral structure TMV virus by binding to the coat protein of the virus, thus weakening its infectivity and infectivity (Zhang J. et al., 2021).

### 2.7 Esters or Lactones Derivatives

**FIGURE 14:**
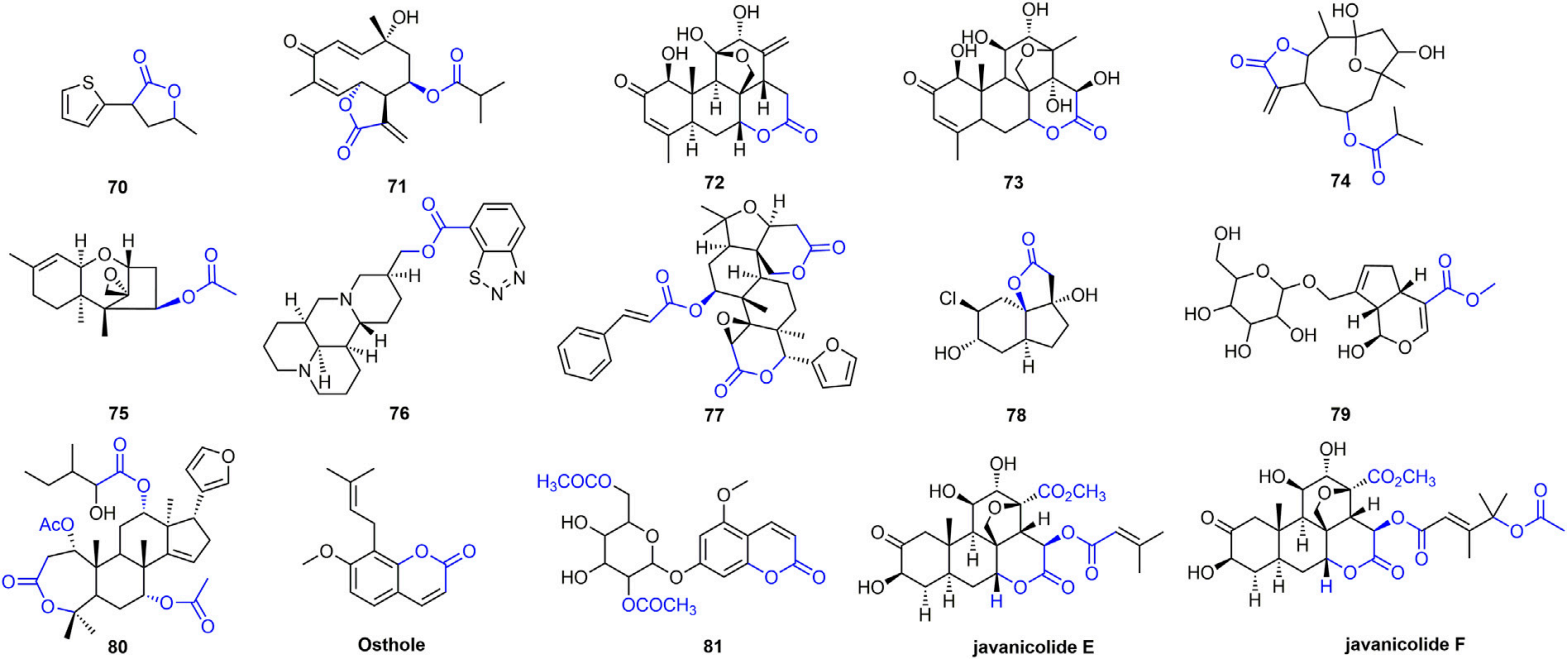
Structures of representative ester or lactide derivatives in antiviral activities.

Esters or lactones have made great progress in the study of antiviral activity, which has attracted the attention of researchers (Olivon et al., 2015; Eriksson et al., 2008; Hellwig et al., 2003). At 500 mg/L, the inactivating, curative, and protective activities of compound **70** (Figure 14) were 52, 57, and 56%, respectively (Lu et al., 2014). The curative activity of compound **71** against TMV was 62.86% at 100 mg/L, and it could inhibit TMV infection by interfering with the expression of TMV-CP (Zhao et al., 2017c). Compound **72** inhibits the expression of tobacco TMV-CP with an IC_50_ value of 5.56 *μ*M (Tan et al., 2018; Yan et al., 2010). Compound **73** has obvious inhibitory activity against TMV infection and replication with IC_50_ of 13.98 and 7.13 mg/L, respectively (Shen et al., 2008). Compound **74** inhibited gene expression of TSWV by more than 85%. Compound **74** activates the JA pathway, promotes PAL activity, induces systemic resistance, inhibits gene expression of TSWV, and defends against TSWV infection (Zhao et al., 2020b). Antiviral activity of other representative ester or lactone derivatives are shown in Table 3.

**TABLE 3:** Antiviral activity of other representative ester or lactone derivatives.

Comp	Anti-TMV Activity				Mechanism	Ref
	Concentration (mg/L)	Curative (%)	Protective (%)	Inactivating (%)		
75	—	—	—	—	Compound **75** can delay transmission of pepper mottle virus (PepMoV) in host plants and protect host plants from PepMoV infection	(Ryu et al., 2017)
76	500	72.3	75.7	76.9	—	(Ni et al., 2017)
77	500	41.7	29.3	65.7	—	(Huang et al., 2020)
78	500	-	-	98	—	(Zhu et al., 2019)
79	500	45.8	39.4	42.9	—	(Xia et al., 2018)
80	500	—	—	—	Can inhibit the accumulation of TMV CP *in vitro*	(Ge et al., 2012)
81	—	—	—	—	Inhibiting viral replication	(Zhang et al., 2018)
Osthole	500	70.2	61.9	—	The epidermal protein levels of TMV were significantly reduced, and its replication might be inhibited	(Chen et al., 2020c)

### 2.8 Berberine Analogs

**FIGURE 15:**
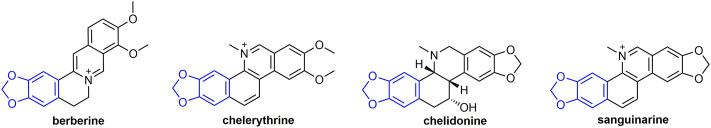
Structures of representative berberine analogs in antiviral activities.

The protective activity of berberine (Figure 15) against TMV was 62.8%. Berberine induces an immune response to TMV in tobacco and associated with systemic resistance through activation of salicylic acid signaling (Guo et al., 2020). At 500 mg/L, chelerythrine had obvious inactivation, proliferation inhibition, and protection effects on TMV, and the inhibition ratio were 72.67, 77.52, and 59.34%, respectively. (Guo et al., 2021).

## 3 Conclusion

In recent years, great progress has been made in the research and development of antiviral agents. The discovery of some new antiviral agents has provided more options for the prevention and control of plant virus diseases. These new antiviral agents are expected to become pillar products in the future. These novel antiviral agents are obtained by structural modification of natural products as lead compounds. In the past 10 years, some important natural products or backbone structures based on natural products in the research and development of anti-plant virus agents are mainly acids (fatty acids, carboxylic acids, and ferulic acids), ketones (chalcones, pentadienones, quinazolinones, and chromones), vanilloids, indoles, silmenines, purine nucleosides, esters or lactones, and berberine analogs. Among them, the derivatives based on vanillin and indole show great application prospects. For example, xiangcaoliusuobingmi and fubianliusuoyoumi are novel antiviral agents based on vanillin, and chloroinconazide is a novel antiviral agent based on indole. Currently, the discovery methods of anti-plant viral agents mainly include natural product isolation, natural product-based structural modification, protein-based structural design, and computational-based structural optimization. The research on the mechanism of action of antiviral agents mainly focuses on the relationship between drugs and RNA, proteins, and pathways, and the in-depth mechanism of action in living host plants needs to be further explored. The design of new scaffolds and lead compounds inspired by natural products has played an important role in the development of antiviral agents and has been demonstrated in practice. With the continuous discovery of new natural products, more anti-plant virus agents based on natural products will be discovered and applied in the future.
